# Noninvasive measurement of transdermal drug delivery by impedance spectroscopy

**DOI:** 10.1038/srep44647

**Published:** 2017-03-24

**Authors:** Pasquale Arpaia, Umberto Cesaro, Nicola Moccaldi

**Affiliations:** 1 University of Naples Federico II, Department of Electrical Engineering and Information Technology, Via Claudio21, 80125 Naples, Italy; 2 CRdC Tecnologie Scarl, Via Nuova Agnano, 11-80125 Naples, Italy

## Abstract

The effectiveness in transdermal delivery of skin permeation strategies (e.g., chemical enhancers, vesicular carrier systems, sonophoresis, iontophoresis, and electroporation) is poorly investigated outside of laboratory. In therapeutic application, the lack of recognized techniques for measuring the actually-released drug affects the scientific concept itself of dosage for topically- and transdermally-delivered drugs. Here we prove the suitability of impedance measurement for assessing the amount of drug penetrated into the skin after transdermal delivery. In particular, the measured amount of drug depends linearly on the impedance magnitude variation normalized to the pre-treated value. Three experimental campaigns, based on the electrical analysis of the biological tissue behavior due to the drug delivery, are reported: (i) laboratory emulation on eggplants, (ii) *ex-vivo* tests on pig ears, and finally (iii) *in-vivo* tests on human volunteers. Results point out that the amount of delivered drug can be assessed by reasonable metrological performance through a unique measurement of the impedance magnitude at one single frequency. In particular, *in-vivo* results point out sensitivity of 23 ml^−1^, repeatability of 0.3%, non-linearity of 3.3%, and accuracy of 5.7%. Finally, the measurement resolution of 0.20 ml is compatible with clinical administration standards.

Topical and transdermal drugs are employed with the twofold objectives of minimizing the systemic uptake and concentrating the action at the site within the skin^1^. Topical administration and transdermal delivery are advantageous in comparison with systemic administration routes because complications as first-pass metabolism, toxicity, and side effects are attenuated for the patient^2^.

In transdermal delivery, different strategies are used to facilitate skin permeation: chemical enhancers, vesicular carrier systems, sonophoresis, iontophoresis, and electroporation^3^,^4^. Their effectiveness is evaluated by numerous studies in laboratory, surveyed in^5^,^6^. However, performance outside of a laboratory, in the context of daily clinical application, is less investigated^6^. The absence of effective and standardized measurement methods *in vivo* for locally acting drugs makes problematic the scientific use of the concept itself of dosage for topical and transdermal delivery^7^.

At present, some methods allow precise measurements but are invasive and not immediate. In case of skin biopsies, for example, whether at the dermis level (“shave biopsy”), or through the subcutis (“punch biopsy”), local anesthesia is to be practiced. For “suction blisters”, a partial negative pressure applied to the skin disrupts the epidermal–dermal junction, and forms a blister filled progressively with interstitial fluid and serum. A previously-applied drug can be sampled in the blister by a hypodermic needle and quantified. Others methods are less invasive, but hard to standardize^6^, e.g., tape stripping and microdialysis. In particular, tape stripping involves the sequential removal of microscopic layers of stratum corneum. Usually, an adhesive tape strip is placed initially onto the skin surface by a gentle pressure to ensure a good contact, and subsequently removed by a sharp upward movement. Microdialysis requires the insertion of a small catheter with a semipermeable hollow fiber membrane at its tip. After the insertion, small solutes can cross the semipermeable membrane by passive diffusion. Other recent and promising methods are Confocal Raman spectroscopy and the colorimetry. The former couples a Raman spectrometer to a standard optical microscope, allowing high-magnified visualization of a sample and Raman analysis by a microscopic laser spot. However, very-expensive equipment is needed, and moreover only a direct analysis of the most superficial skin layers is provided^8^. For corticosteroids, a colorimetric scale measures the vaso-constrictive effect of the active principle through the whitening extent of the treated tissues^9^. However, the measurement is still inaccurate and burdensome, thus not easily transferable in daily clinical application.

Conversely, the measurement of bioimpedance is widely considered as a very-low invasive investigation method, extensively exploited both in biomedical research in general, and particularly also in dermatology: to limit reproducibility problems in the context of *ex*–*vivo* penetration studies for topical applied drugs^10^; to measure variations of the hydration state of the horny layer^11^, and, in this way, to predict its permeability^12^; to differentiate the interaction of diverse pharmacological principles with the skin tissue^13^; to allow early detection of pressure ulcers *in vivo*^14^; to measure the blood flow caused by treatment heat transfer^15^; and, last but not least, for cancer diagnosis^16^. As a first approximation, a tissue can be considered as an electrolyte containing densely packed cells. In conductometry, the variation in the measured resistance of a solution due to addition of a conductive substance allows the dissolved amount to be tracked (the equivalent conductance varies according to the added substance). Analogously, in a biological tissue, characterized by ionic conductivity and dielectric relaxation phenomena, the impedance measurement provides useful information to assess the injected amount of a conductive substance, once the equivalent impedance of the tissue was measured before injection.

In this paper, the amount of a substance delivered in a tissue is assessed through a impedance variation measurement, once the relationship between this variation and the delivered amount is known. Measurement of impedance variation normalized to pre-delivered value is proven to be suitable for assessing the amount of drug penetrated into the skin after transdermal delivery treatment *in vivo* application. To this aim, three experimental campaigns, based on the analysis of the biological tissue electrical reaction to the drug delivery, are reported: (i) *laboratory tests* of emulation on eggplants, (ii) *ex-vivo tests* on pig ears, and (iii) *in-vivo tests* on human volunteers.

## Laboratory tests

The behavior of the equivalent impedance spectrum of an eggplant pulp volume at varying the injected drug amount was investigated in laboratory, under controlled conditions. In particular, the tests were aimed at:

- Proving in principle the *feasibility* of the impedance-based assessment method.
- *Validating* the experimental procedure.
- Carrying out a *metrological characterization*.
- Determining the *optimum metrological performance*.

In these tests, as usual for laboratory experiments of dermatological topical treatments, the eggplant was chosen among different vegetables and fruits already characterized by impedance spectroscopy investigations (potato, tomato, kiwi, orange, and so on) for twofold main reasons. Firstly, the eggplant tissue has a significant capability of emulating the skin electrical behavior. In fact, its tissue is modeled by an equivalent electrical circuit with concentrated parameters similar to those of widely-used electrical models of the human tissue^17^,^18^. Secondly, the eggplant was selected owing to its porosity and consequent capability to be easily penetrated by a needle in a calibrated way. For the potato, any attempt of injection even by insulin needle, also of the order of 100 *μl*, determines the immediate spillage. Thus, injecting drugs into potato was considered as not feasible. Analogously, the fruit was excluded: the excessive presence of liquids produces ample channels to the ionic conductivity mechanism (ohmic), and significantly flattens the curve of the impedance spectrum more than other biological tissues^19^,^20^.

### Feasibility

A screening measurement campaign was aimed at proving the feasibility of the assessment method and identifying the bandwidth of interest. In the following, (*i*) the *measurement setup*, and (*ii*) the *experimental results* are detailed.

#### Set up

Seven rectangular-shaped pulp sample blocks, each of 40 × 40 × 100 mm, were derived from as many eggplants. Impedance spectroscopy was carried out at room temperature by using a Solartron 1260A Impedance Analyzer (Solartron Analytical, Hampshire, UK). A two-terminal configuration was selected owing to the negligible effect of parasitic capacitance and to the expected medium impedance levels in the interest bandwidth of few MHz. Pre-gelled electrodes with one of the largest surfaces on the market (7.28 cm^2^) were selected in order to mitigate the impact of the contact on the measurement. The electrodes PG 500 FIAB, with conductive gel allowing a good adhesion to the surface, specific for bio-impedance analysis, were used. Electrodes gap was 4.6 cm. A commercial drug with a conductivity of 526 *μ*S/cm (water, sodium acrylates copolymer, hyrogated polydecene, PPG-1Trideceth-6, phosphatidylcnoline, urea sorbitol, glycerin, butylene glycol) was injected 2 cm below the surface for the following volumes: 0.0, 0.4, 0.8, and 1.2 ml. A sinusoidal current of 1 mA was applied to the electrodes. The voltage drop was measured in the frequency range of 1 Hz to 10 MHz by a logarithmic frequency sweep of 10 steps/decade. After the preparation of the eggplant and the electrode application, the impedance spectrum was measured before the injection set and after each injection level.

#### Results

**Figure 1:**
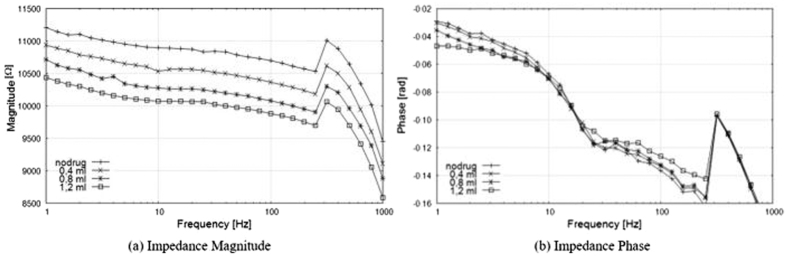
Impedance of eggplant pulp at varying the injected amount: Bode diagrams.

In the above experimental conditions, a linear relationship between amount of injected substance and impedance spectrum was surveyed. In particular, the typical Bode diagrams in Fig. 1a highlight an increase in the injected amount of drug determining a related progressive decrease in the impedance magnitude, whilst (Fig. 1b) the trend of the phase does not seem to be analogously regular. These observations are valid for the entire frequency range at varying the experimental conditions (different eggplant, electrode area, electrode gap, signal amplitude, signal frequency, as well as differently conducting drugs) around the abovementioned values. The fluctuation of impedance and phase in the range of 150–1000 Hz in Fig. 1 arises from the error sources acting usually on biomedical measurements by impedance spectroscopy in dermatological applications, and namely mainly from the electrode/electrolyte interface^21^. The electrical impedance of intact human skin is dominated by the stratum corneum at low frequencies and electrode/electrolyte interface effects are negligible. In case of a peeled eggplant, the uncertainty sources are not masked by a stratum corneum. Anyway, the effect is deterministic and does not affect significantly the highlighted relationship between amount of drug and impedance magnitude.

According to these results, a simple impedance measurement at a single frequency can be used to determine the drug amount instead of a spectrum analysis over the bandwidth as a whole.

### Validation

The relationship between the amount of injected substance and the variation in the impedance magnitude was determined experimentally. The percentage variation from the initial impedance magnitude was measured in order to soften the impact of the initial conditions and common mode noise. Moreover, this allows to abstract from the specific bio-dynamics of the skin by improving measurement reproducibility at varying the individual and interindividual skin characteristics.

#### Set up

Rectangular-shaped pulp samples of 40 × 40 × 100 mm were cut from the central part of an eggplant fruit. For each sample, the same preparation time between peeling, electrodes application, and measurement was ensured. Drug was injected 2 cm below the surface for the following volumes: 0.0, 0.2, 0.4, 0.6, 0.8, and 1.0 ml. Measurements were carried out by the Agilent 4263B LCR Meter, in the following conditions Table 1 - second row: (*i*) signal amplitude of 20 mV to pursue linearity; (*ii*) 1 kHz signal frequency to mitigate electrode influence (more significant at low frequency), (*iii*) electrode gap referring to the ideal condition of current depth within the tissue roughly half the electrode distance, and (*iv*) average on 60 repetitions, to filter random noise.

#### Results

**Figure 2:**
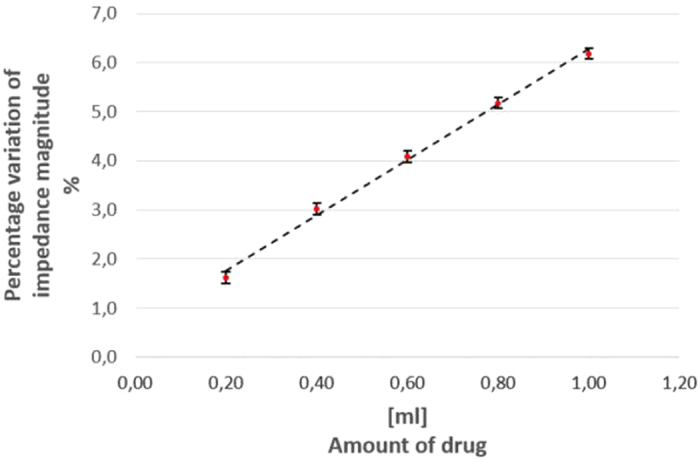
Impedance magnitude percentage variation vs drug amount in laboratory experiments.

The typical linear behavior between percentage variation of impedance magnitude and drug amount, pointed out in Fig. 2, was experienced. In particular, for a drug with higher conductivity than the eggplant, by increasing the drug level, the measured equivalent impedance decreases accordingly. Furthermore, once normalized to the pre-injection value, a linear relationship between injected level and percentage impedance variation is achieved.

### Metrological characterization

The experimental quality of the assessment method was tested in order to verify preliminarily the impact of the *drift*, and then to compute *sensitivity, nonlinearity repeatability, accuracy*, and *resolution*.

#### Drift analysis

Among the error sources mostly affecting the measurement of a constant drug amount, the drift plays a prominent role. Drift^22^ arises mainly from twofold main sources: (*i) the evaporation of the water contained within the eggplant*, mainly participating in the creation of conductive channels for ions, and thus increasing the impedance; and (*ii) the electrodes degradation*, due to the gradual penetration of the electrode gel within the eggplant^23^: the high concentration of ions in the electrolyte gel participates in the conduction process significantly.

**Figure 3:**
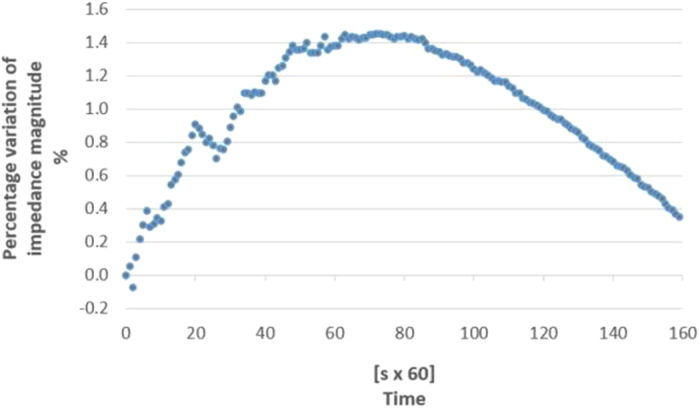
Measurement drift due to eggplant evaporation and electrode gel permeation.

Therefore, a specific experimental analysis was aimed at assessing the drift impact on the measured impedance. The observation on 5 samples for a time interval of 10^4^ s led to results in Fig. 3.

The drift is predominated initially by the effect of the gel penetration on the eggplant evaporation. As a matter of fact, the equivalent impedance magnitude is decreasing because the gel penetration increases the conductivity. The situation is reversed about after half an hour, when the impedance begins to increase, because the water decrease leads to a conductivity increment. Upon 15 minutes after the electrodes application, the drift effect is limited to well below 1% of the initial impedance, namely 0.4%.

A typical measurement protocol includes the progressive administration of 5 doses and lasts 3 minutes in total from the electrodes application. A single impedance measurement is carried out in 1 s. After each increase in the injected drug, 10 impedance measurements are carried out for a total of 10 s. The 10 results are then processed by computing average and standard deviation. The time left to the technician to administer the new dose is 10 s. Therefore, the drift does not affect the measurement accuracy.

#### Sensitivity

The sensitivity of the assessment method was determined as the slope of the above linear regression model: 3.8 ml^−1^. In particular, for a typical dose of 10 ml of injected drug, a corresponding percentage variation of 38% in impedance magnitude with respect to the reference value of the pre-injection tissue can be achieved.

#### Nonlinearity

The specification about sensitivity is funded on the assumption of a linear model. The error of such an assumption was assessed by one-way ANalysis Of VAriance (ANOVA), as the residual standard deviation of the linear regression model (nonlinearity). A typical value of 0.47% was achieved.

#### Repeatability

**Figure 4:**
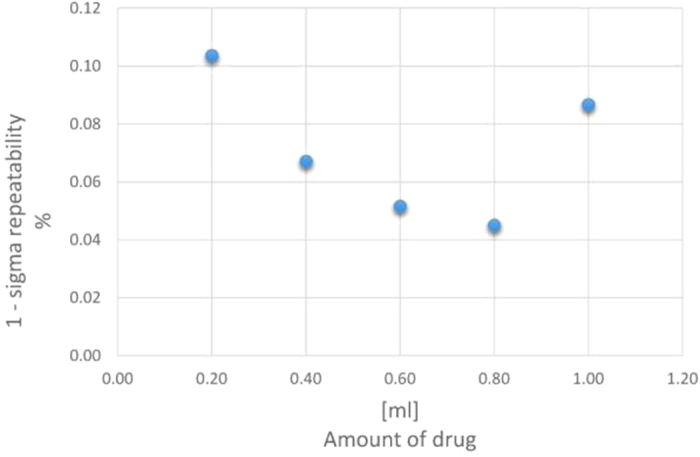
1-sigma repeatability vs the amount of injected drug.

In Fig. 4, the 1-sigma repeatability, expressed as relative percentage with respect to the initial impedance value, is plotted as a function of the amount of injected drug. A typical value of 0.07% was determined.

#### Accuracy

**Figure 5:**
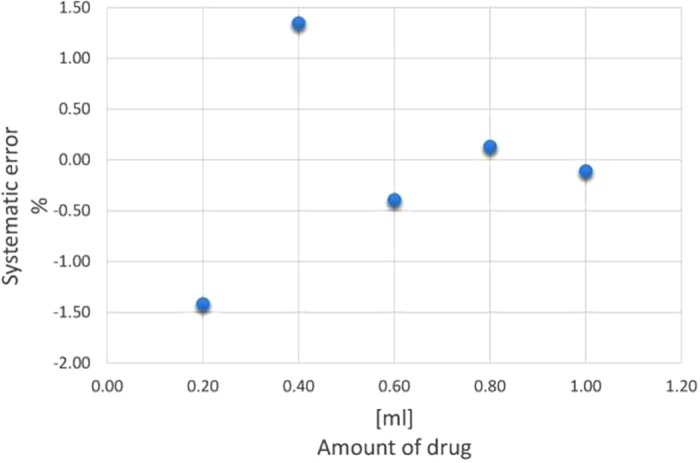
Percentage deterministic error vs the amount of injected drug.

In Fig. 5, the percentage deterministic error is plotted as a function of the injected drug. A typical value of accuracy of less than few percent is obtained by averaging the maximum values. This error can be compensated by computing its value for a given measurement configuration (calibration).

#### Resolution

The resolution was computed as the indetermination in the minimum measurable amount of drug, in terms of the uncertainty of the linear regression model. In particular, the mean squared error of the single value predicted by ANOVA was computed.

The metrological characteristics of the assessment method are summarized in the last row of Table 1.

### Performance optimization

In order to enhance the method sensitivity, while maintaining satisfying levels of non-linearity, repeatability, and resolution, main significant parameters of the experiment were investigated. In particular, the electrode surface and gaps are directly related to the amplitude of the investigated volume and location of the maximum concentration of the generated current. The drug conductivity and the signal amplitude are assumed directly related to the sensitivity while the frequency of the signal should be characterized by an inverse relationship with the sensitivity considering the contribution by the substance injected mainly ohmic. Three possible electrode surface levels were identified as fractions (1.00, 0.50, 0.25) of the original surface, in order to facilitate the realization through the cut. The maximum distance on the specimen surface of the eggplant for two electrodes of maximum dimensions was identified equal to 4.60 cm. This value is derived from the difference between the length of the specimen (10 cm) and the sum of the lengths of the two electrodes (27 × 2 mm). Two further experimentation values were obtained by 1.60 cm step up a minimum of 1.40 cm, necessary to allow the infiltration of the drug. The amplitude levels and the stimulus signal frequency were identified using all the range allowed by commercially available instruments (e.g., the LCR Meter Agilent 4263B). For the drug conductivity, the same solution used in the previous tests was exploited to test a conductivity variation of about 25% (from 526 to 666 *μ*S/cm).

The influence of the parameters on the metrological characteristics was investigated by means of a statistical parameter design, based on a Taguchi experimental plan L18^24^. At this aim, 18 rectangular-shaped pulp sample blocks, each of 40 × 40 × 100 mm were cut from the central part of an eggplant. For each sample, the same preparation time between peeling, electrodes application and measurement, was observed. Drug was injected 2 cm below the surface at the following amounts: (0.0, 0.2, 0.4, 0.6, 0.8, 1.0) ml. Measurements were carried out by Agilent 4263B LCR Meter. In the *i-th* experiment of the plan L18, the measurement setup was configured according to the combination of parameters levels corresponding to the *i-th* matrix row^25^, such as pointed out in Table 2. In each experiment, the percentage variation from the initial impedance magnitude was computed for all the injected drug volumes. Each experiment was repeated 60 times, 10 for each of the 6 levels of injected drug, by computing sensitivity [ml^−1^], repeatability [%], nonlinearity [%], and resolution [ml] (Table 2).

**Figure 6:**
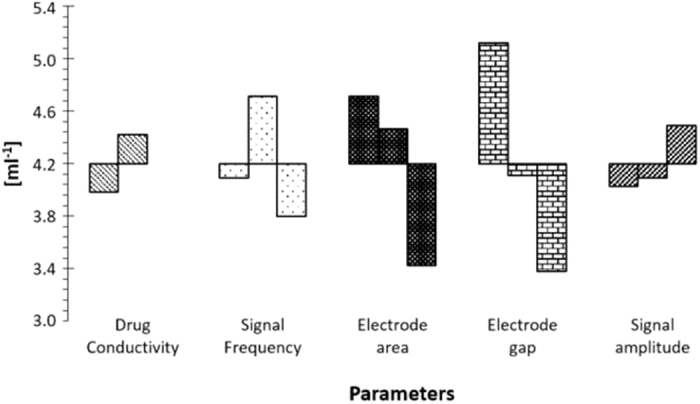
Influence of the experiment parameters on the method sensitivity.

The mean over the 18 experiments of each metrological characteristic is pointed out in Table 2. In Fig. 6, the influence of the experiment parameters on the method sensitivity, computed by Analysis of Mean (ANOM)^26^, is highlighted.

**Figure 7:**
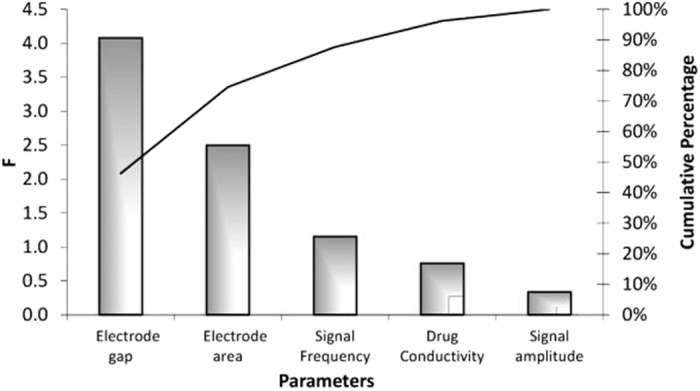
Ranking of parameters influence on the sensitivity.

By ANOVA, the statistical significance of the incidence of each parameter on the measurement sensitivity is assessed. The variance ratio Fi (usually referred to also as F-statistic^26^) was computed as the ratio between the sensitivity variance due to the *i*–*th* parameter and the error variance. The histogram of Fig. 7 (Pareto diagram^26^) highlights that the electrode gap is the most influencing parameter, followed in order by the electrode area, signal frequency, drug conductivity, and signal amplitude. The optimum configuration is predicted by means of the design parameter effects maximizing the sensitivity by referring to the histogram of Fig. 6: drug conductivity 666 *μ*S/cm, signal frequency 1.0 kHz, electrodes area 1.82 cm^2^, electrodes gap 1.4 cm, and signal amplitude 1.0 V.

It is worth to note that an increase by about 25% in drug conductivity produces a corresponding sensitivity improvement of about 12%.

## *Ex-vivo* tests

In *ex-vivo* tests, according to literature^27^,^28^, pig skin appeared as the most suitable model for human skin. Several properties of porcine and human skins (e.g., epidermal thickness and lipid composition), as well as the permeability of the membranes to diverse compounds, are significantly similar^29^.

Three ears of domestic pigs, with less than few hours postmortem, were acquired from a local abattoir. Dead cells of the stratum corneum were removed by first placing onto the skin, and then removing by a single movement, a 5 × 5 cm adhesive tape, for 20 times. The same configuration setup optimized for laboratory tests was employed but, in this case, the injection depth was reduced to 0.5 cm, by injecting the same solution.

**Figure 8:**
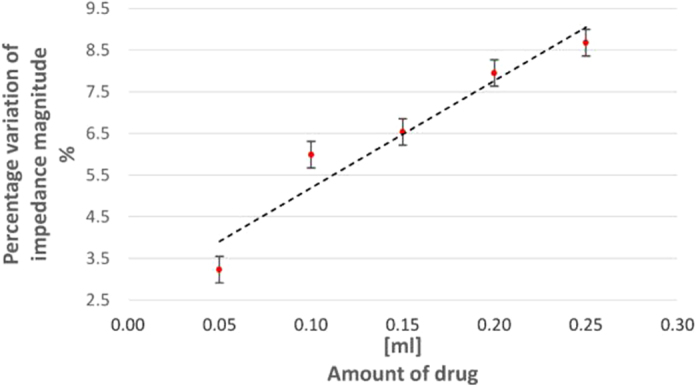
Impedance magnitude percentage variation vs drug amount in *ex-vivo* experiments.

Analogously as for the laboratory experiments, a linear trend was experienced (Fig. 8). In Table 3, the obtained metrological characteristics of *ex-vivo* tests are compared with laboratory performance in the same conditions: (*i*) sensitivity increases considerably owing to the reduced injection depth; (*ii*) higher nonlinearity is detected (5.04% with respect to red 3.64%); (*iii*) 1 − σ repeatability decreases from 0.11% to 0.47%, owing to the higher variability in electrical response of pig cells with respect to eggplant; (*iv*) the accuracy worsens from 4.38 to 6.20%, owing to the lower average reproducibility intrinsic to *ex-vivo* experiments; and (*v*) analogously, also the resolution worsens from 0.35 to 0.44 ml.

## *In-vivo* tests

**Figure 9:**
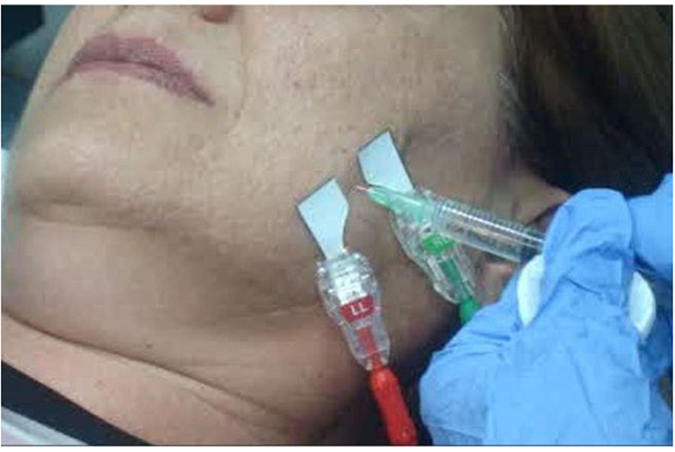
Drug injection during *in-vivo* experiments.

*In-vivo* experiments were carried out in an authorized Institute on subjects of different ages and sex (Fig. 9), which gave their informed consent to participate to publish the related information and images in an on-line open-access publication. Measurements were carried out by means of an instrument^30^ specifically prototyped for *in-vivo* use, in compliance with safety regulations. All methods were carried out in accordance with relevant guidelines and regulations. All experimental protocols were approved by IMPALab and IRCCS FP ethical committees.

Experimental tests highlighted that the set-up design achieves maximum accuracy and repeatability in case of 520 mV stimulus signal level for analogous *ex-vivo* test conditions. A water soluble sodium salt of hyaluronic acid, with conductivity of 1.37 mS/cm, was used. The depth of injection was 0.5 cm below the surface, such as in ordinary cosmetic treatments. In synthesis, measurement conditions were: drug conductivity = 1.37 mS/cm; signal frequency = 1.00 kHz; electrodes area = 1.82 cm^2^; electrode gap = 1.4 cm; and signal amplitude = 520 mV.

**Figure 10:**
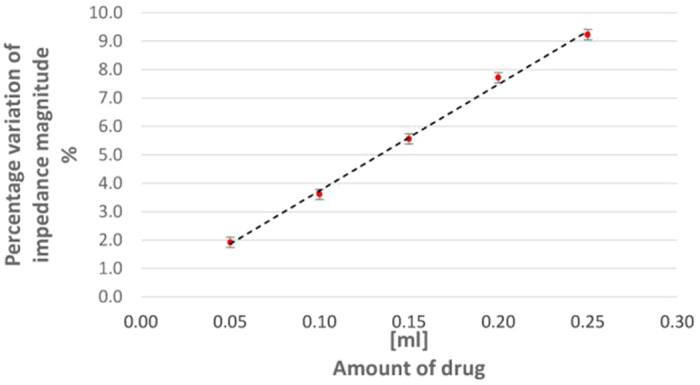
Impedance magnitude percentage variation vs drug amount in *in-vivo* experiments.

The experiments were repeated for 10 times on 8 subjects for the following volumes of injected drug: 0.0, 0.05, 0.10, 0.15, 0.20, and 0.25 ml. A linear behavior was again found, as pointed out in Fig. 10. Metrological characteristics of *in-vivo* tests are compared with laboratory, and *ex-vivo* performance in Table 4 for the same setup configuration, drug and sample size (8): (*i*) sensitivity is comparable, although decreased with respect to values in Table 3, owing to the higher viscosity of the solution injected in *in-vivo* tests; (*ii*) nonlinearity is lower than in *ex-vivo* tests (3.31% vs 4.25%), though still slightly higher than in laboratory (2.35%); (*iii*) 1 − *σ* repeatability slightly worsens from 0.16% to 0.27% (though still again worse than in laboratory, 0.07%); (*iv*) the accuracy improves from 7.40 to 5.71%, and is still compatible with laboratory (5.23%); and (*v*) analogously, also the resolution improves from 0.37 to 0.19 ml, even higher than in laboratory (0.23 ml).

Such results highlight the suitability of impedance measurement for assessing the amount of drug actually penetrated into the skin after *in-vivo* treatment.

## Conclusions

For a biological tissue and in particular for human skin, a linear relationship between injected drug and the variation of the normalized impedance, measured at a frequency of 1 kHz, has been found. The resolution is still compatible with the standard of dermatological administration. The use of substances with electrical conductivity of the order of mS/cm allows to reach high levels of sensitivity: for volumes of 10 ml of product, the normalized impedance magnitude variation could be higher than 100%. The sensitivity is related directly to the drug conductivity: for the eggplant, an increase by about 15% in conductivity produces a corresponding sensitivity improvement of about 15%. The *in-vivo* measuring system also exhibits significant sensitivity of about 23 ml^−1^, repeatability of 0.3%, non-linearity of 3.3%, and accuracy of 5.7%, still comparable with more-controllable laboratory conditions. A stand-alone instrument, based on the principle experimented here, is in an advanced realization stage in order to be used for drug delivery experimentation in clinical environment.

The dispersion due to interindividual reproducibility may be too large for the desired uncertainty in case of clinical applications. The use of impedance spectroscopy, introducing additional degrees of freedom than the mere impedance, could allow the calibration curve (impedance vs drug) to be determined for each treated tissue, bringing back the reproducibility at acceptable values. This will open interesting scenarios to pursue strategies of immediate efficacy assessment for all non-invasive systems for intradermal drug administration.

## Additional Information

**How to cite this article:** Arpaia, P. *et al*. Noninvasive measurement of transdermal drug delivery by impedance spectroscopy. *Sci. Rep.* **7**, 44647; doi: 10.1038/srep44647 (2017).

**Publisher's note:** Springer Nature remains neutral with regard to jurisdictional claims in published maps and institutional affiliations.

## Figures and Tables

**Table 1:** Metrological condition and result in the metrological characterization.

Parameter	Drug Conductivity	Signal Frequency	Electrodes Area	Electrodes Gap	Signal Amplitude
Value	666 *μ*S/cm	1.00 kHz	3.64 cm^2^	4.6 cm	20 mV
**Characteristic**	**Sensitivity [ml^−1^]**	**Nonlinearity [%]**	**1 − *σ* Repeatability [%]**	**Accuracy [%]**	**Resolution [ml]**
Value	3.8	0.47	0.07	0.68	0.005

**Table 2:** L18 plan for statistical parameter design in performance optimization of the assessment method.

Run	Parameter					Results			
	Drug Conductivity [*μ*S/cm]	Signal Frequency [kHz]	Electrodes Area [cm^2^]	Electrodes Gap [cm]	Signal Amplitude [V]	Sensitivity [ml^−1^]	Repeatabi-lity [%]	Nonlinea-rity [%]	Resolution [ml]
1	526	0.1	1.82	1.4	0.020	5.6	2.99	2.34	0.130
2	526	0.1	3.64	3.0	0.100	4.7	0.05	0.78	0.036
3	526	0.1	7.28	4.6	1.000	3.0	0.16	3.74	0.115
4	526	1.0	1.82	1.4	0.100	4.7	1.02	4.26	0.197
5	526	1.0	3.64	3.0	1.000	4.3	0.34	3.16	0.140
6	526	1.0	7.28	4.6	0.020	3.1	0.03	3.69	0.118
7	526	10.0	1.82	3.0	0.020	4.3	0.89	2.54	0.107
8	526	10.0	3.64	4.6	0.100	2.7	0.32	3.80	0.098
9	526	10.0	7.28	1.4	1.000	2.9	0.05	2.82	0.083
10	666	0.1	1.82	4.6	1.000	4.0	0.80	1.37	0.055
11	666	0.1	3.64	1.4	0.020	4.4	0.82	1.50	0.067
12	666	0.1	7.28	3.0	0.100	2.4	0.09	7.62	0.190
13	666	1.0	1.82	3.0	1.000	6.0	3.25	0.82	0.049
14	666	1.0	3.64	4.6	0.020	3.8	0.54	0.47	0.018
15	666	1.0	7.28	1.4	0.100	6.4	0.95	2.26	0.113
16	666	10.0	1.82	4.6	0.100	3.6	0.61	1.00	0.036
17	666	10.0	3.64	1.4	1.000	6.7	2.15	0.94	0.062
18	666	10.0	7.28	3.0	0.020	2.5	0.10	2.79	0.073
				Overall mean		4.2	0.84	2.55	0.09

**Table 3:** Metrological characteristics of the impedance-based method for assessing the injected drug.

	Sensitivity [ml^−1^]	Nonlinearity [%]	1 − *σ* Repeatability [%]	Accuracy [%]	Resolution [ml]
Laboratory exp.	30.6	3.64	0.11	4.38	0.35
*Ex-vivo* exp.	34.4	5.04	0.47	6.20	0.44

**Table 4:** Metrological characteristics of the assessment method in laboratory, *ex-vivo*, and *in-vivo* experiments.

	Sensitivity [ml^−1^]	Nonlinearity [%]	1 − *σ* Repeatability [%]	Accuracy [%]	Resolution [ml]
Laboratory experiments	29.5	2.35	0.07	5.23	0.23
*Ex-vivo* experiments	24.5	4.25	0.16	7.40	0.37
*In-vivo* experiments	22.7	3.31	0.27	5.71	0.19
